# Supplementary material for: Clinical knowledge extraction via sparse embedding regression (KESER) with multi-center large scale electronic health record data
Source: NPJ Digit Med. 2021 Oct 27;4:151. doi: 10.1038/s41746-021-00519-z (PMC8551205; doi:10.1038/s41746-021-00519-z)
Supplement: Supplementary file 1 — Supplementary Information [file 41746_2021_519_MOESM1_ESM.pdf]

Supplementary Information of “Clinical Knowledge Extraction via Sparse Embedding Regression (KESER) with Multi-Center Large Scale Electronic Health Record Data”

This supplementary material provides Implementation details of the feature selection approaches, and additional figures and tables.

Supplementary Note 1. Implementation details of the feature selection approaches

As we used dropout training in the feature selection procedures including the local regularized regression and the integrative regularized regression, we up-sampled the original training data with 10 folds to reduce the randomness of the results incurred by dropout.

To select the tuning parameter λ, we split the raw patient-level samples into training and validation sets at each site (11.6:1 split for VA and 1:1 split for MGB), extracted SPPMI matrices for the partitioned data set and factorized them into embedding matrices of the same dimensionality. The derived embeddings are denoted as $\boldsymbol{V}_{train}^{(m)}$ and $\boldsymbol{V}_{valid}^{(m)}$ for the training and validating set at site m, respectively. For each target code w, we specified a candidate set for the tuning parameter that should be broad enough, and learned the coefficients $B_{wc}^{(m)}$'s with the training data $\boldsymbol{V}_{train}^{(m)}$ for each $\lambda \in \Lambda$, using the local or integrative regularized regression as described in (5) or (6) of our paper. Let $\hat{B}_{wc}^{(m)}(\lambda_{m1}, \lambda_{m2})$ be the solution corresponding to $\lambda_{m1}, \lambda_{m2}$ in the site level feature selection, and $\hat{B}_{wc}^{(m)}(\lambda_1, \lambda_2)$ be the solution corresponding to λ_1, λ_2 in the integrative regression.

We then chose the parameters minimizing the sum squared loss:

$$\left\|\boldsymbol{V}_w^{(m)} - \sum_{c \in \Omega_{0.05}^{(m)}} \hat{B}_{wc}^{(m)}(\lambda_{m1}, \lambda_{m2}) \boldsymbol{V}_c^{(m)}\right\|_2^2$$

for each m in the local regression and that minimizes

$$\sum_{m=1}^{M} \left\|\boldsymbol{V}_w^{(m)} - \Sigma_{c \in \Omega_{0.05}^{(m)}} \hat{B}_{wc}^{(m)}(\lambda_1, \lambda_2) \boldsymbol{V}_c^{(m)}\right\|_2^2$$

in the integrative regression. After choosing the optimal parameters, we used it to fit again (5) or (6) with the embedding matrices $\mathbb{V}^{(m)}$'s derived with the full sample, to obtain the final results.

To speed up our tuning procedure, we separately tune the coefficients for the ridge penalty and sparse penalty. First, we fit ridge regression with penalty coefficients λ_2 or λ_{m2} selected by minimizing the sum squared loss on the validation set. Then we take the predicted values output by the tuned ridge regression as a pseudo-outcome to perform sparse or group sparse regression with penalty λ_2 or λ_{m2}, and again select the parameters minimizing the squared loss on the validation set. To tune each lambda, we initially choose the one among

$$10^{-5}, 10^{-5}e^{d}, 10^{-5}e^{2d}, \dots, 10^{-5}e^{1000d},$$

that minimize the sum squared loss on the validation set, where $d = 0.01 ln 10$ so that the candidate set ranges from 10^{-5} to 10^{5}. Denote the selected parameter as λ_{init}. We then choose the parameter among

$$\frac{\lambda_{init}}{4}, \frac{\lambda_{init}}{4} + f\lambda_{init}, \frac{\lambda_{init}}{4} + 2f\lambda_{init}, \dots \frac{\lambda_{init}}{4} + 1000f\lambda_{init},$$

where f =3/800 so that the candidate set ranges from $\frac{\lambda_{init}}{4}$ to $4\lambda_{init}$.

Supplementary Figure 1. Percentage of variation explained by top d-dimensional eigenvectors over a range of d at MGB and VA.

(A) MGB

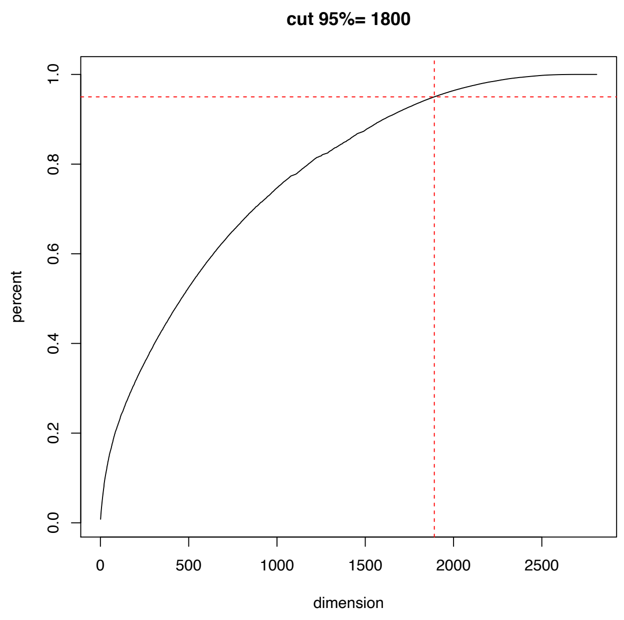

(B) VA.

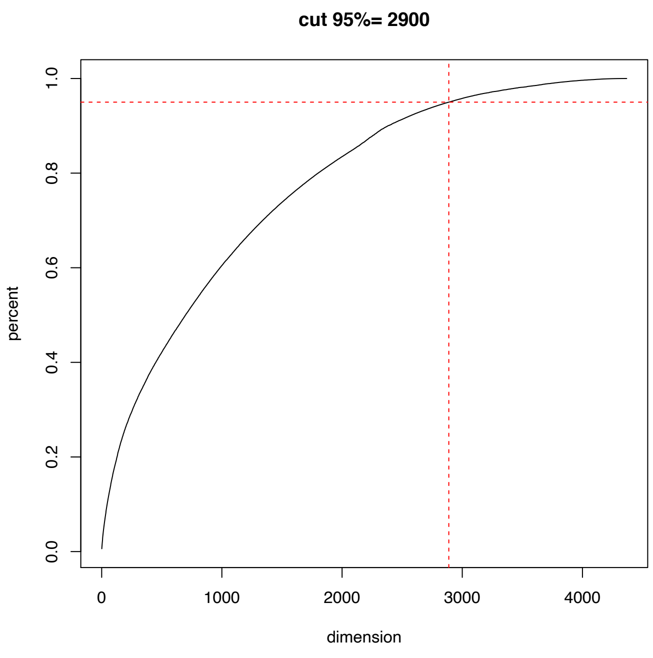

Supplementary Figure 2. KESER$_{VA}$ selected features for Rheumatoid arthritis.

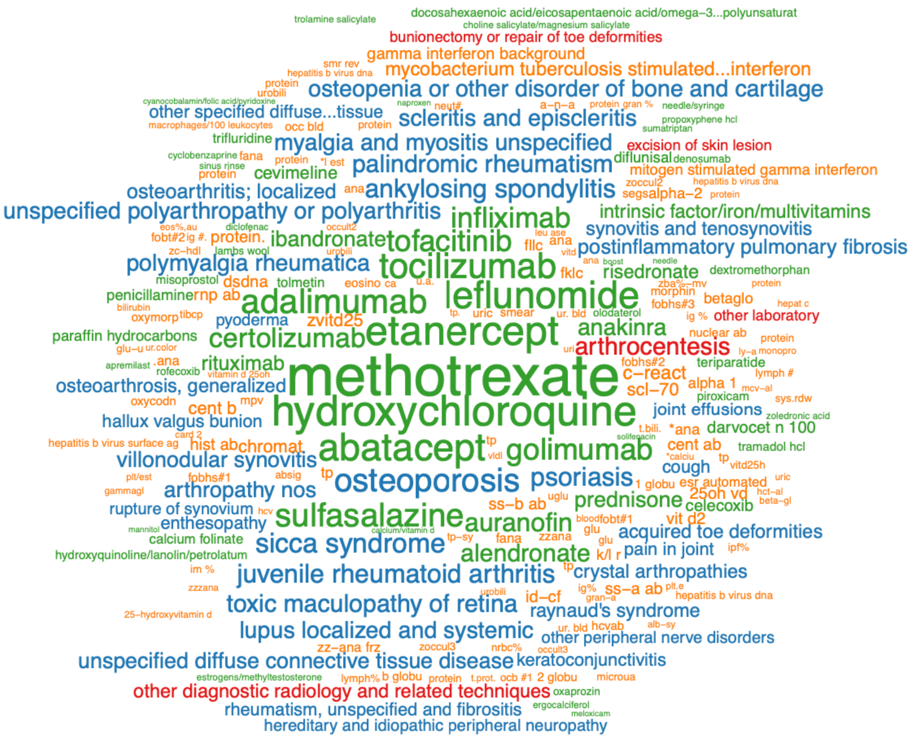

Supplementary Figure 3. KESER selected features for Coronary Artery Disease from MGB and VA.

(A) MGB

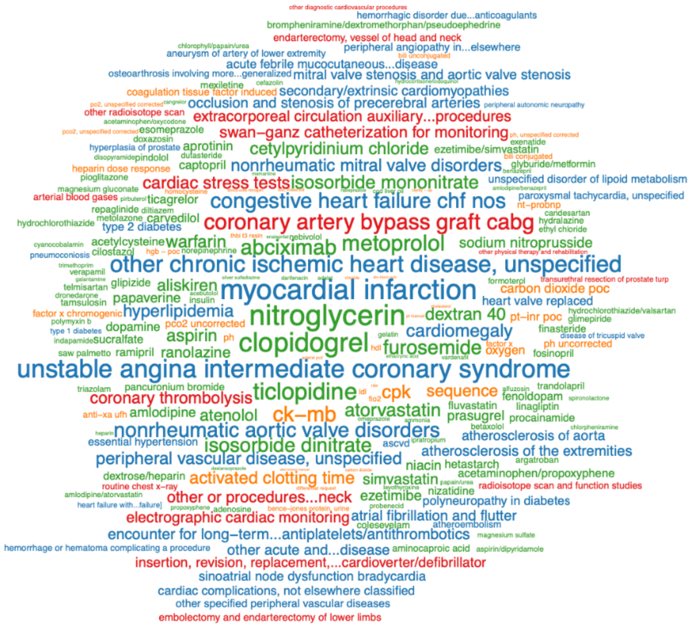

(B) VA

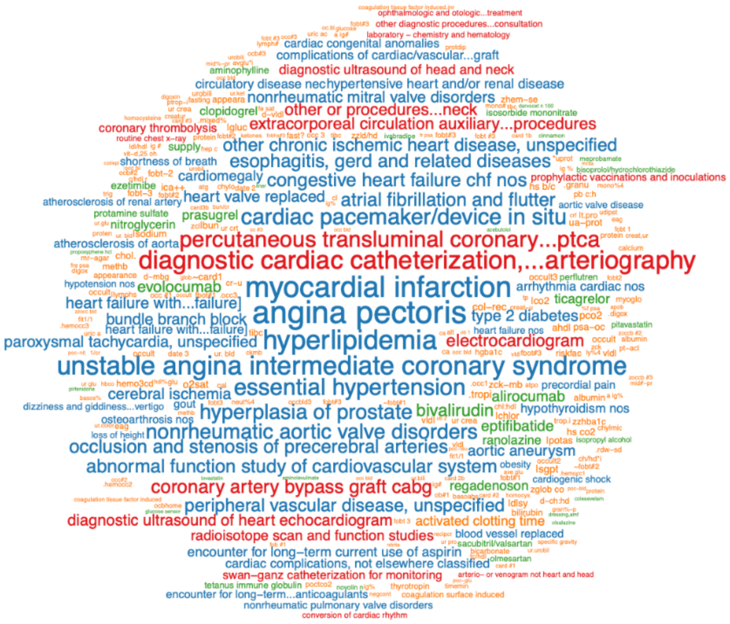

Supplementary Figure 4. KESER selected features for Depression from MGB and VA.

(A) MGB

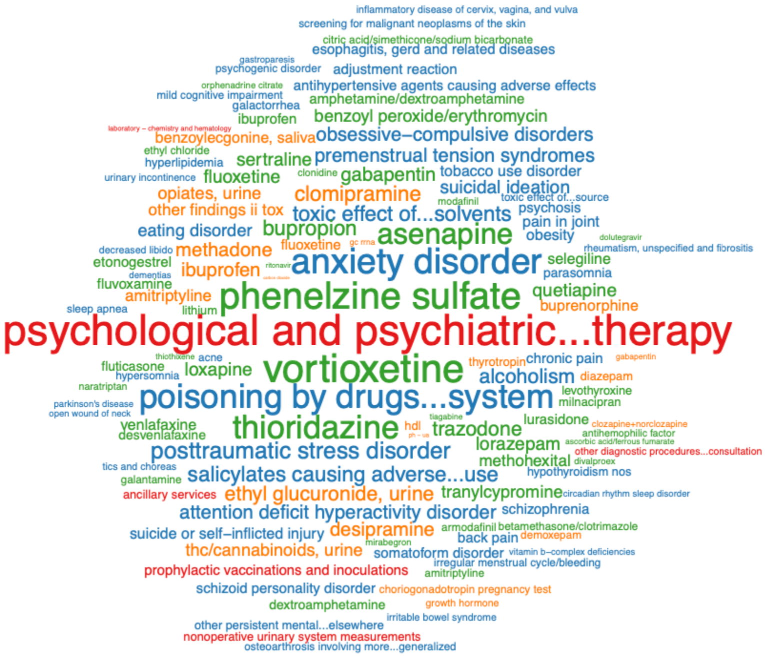

(B) VA

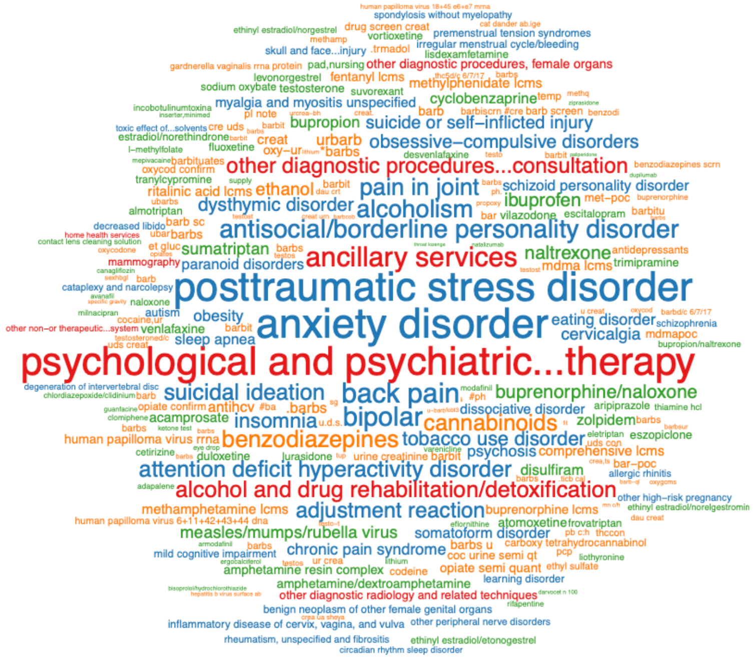

Supplementary Figure 5. KESER selected features for Type 1 Diabetes from MGB and VA.

(A) MGB

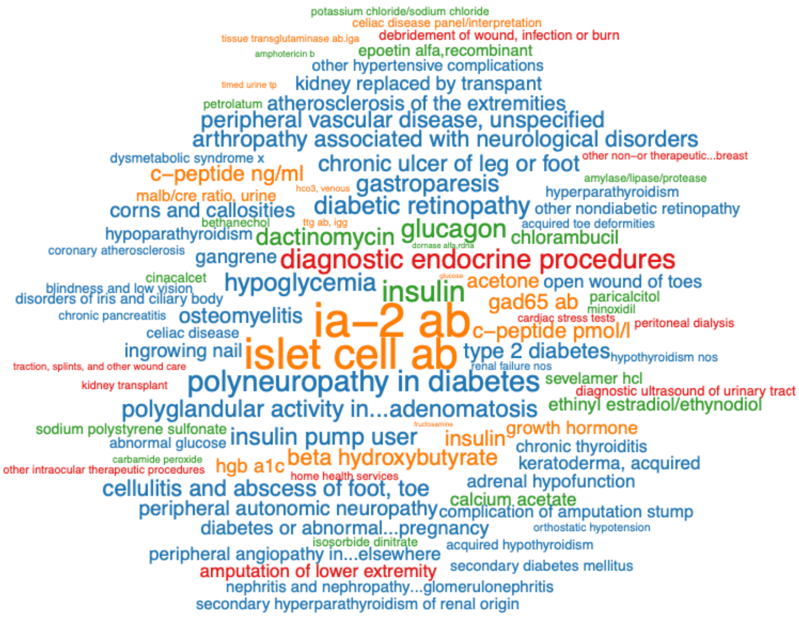

(B) VA

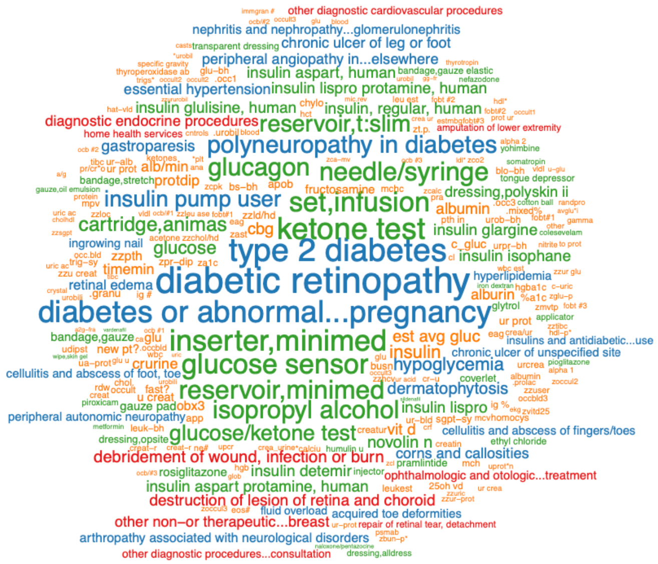

Supplementary Figure 6. KESER selected features for Type 2 Diabetes from MGB and VA.

(A) MGB

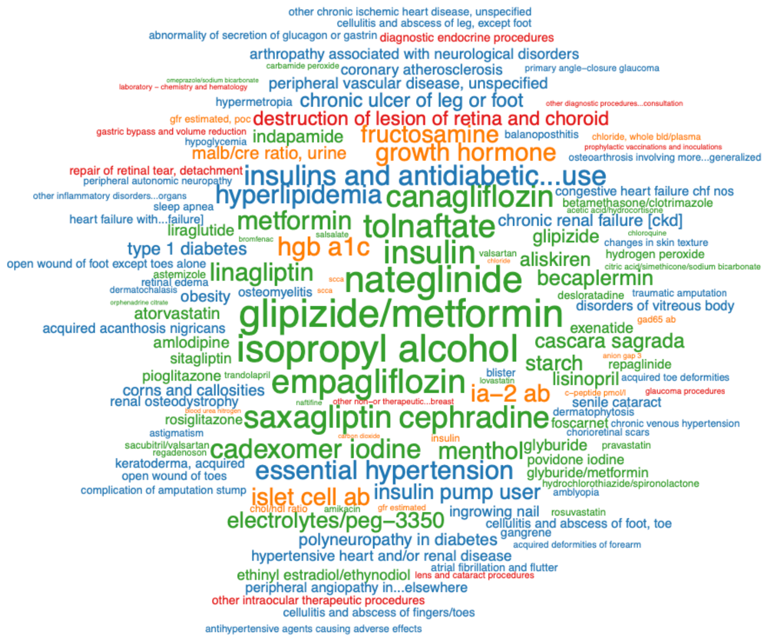

(B) VA

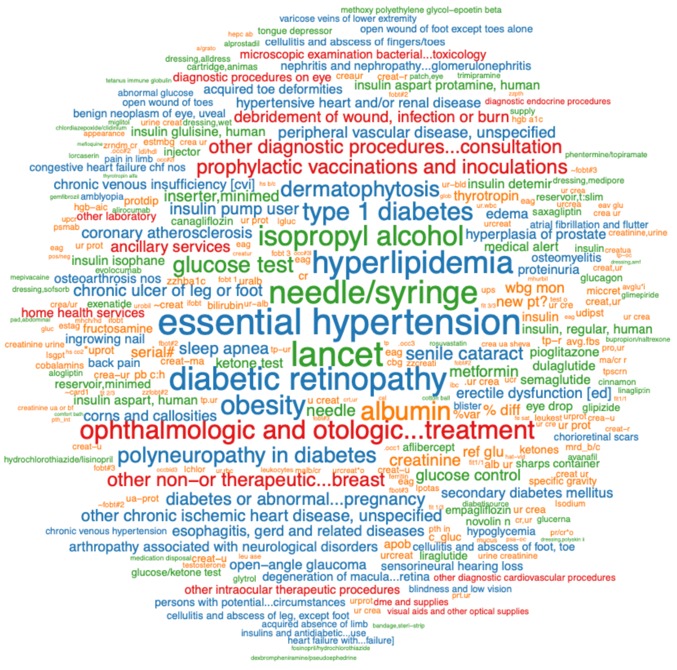

Supplementary Figure 7. KESER selected features for Multiple sclerosis from MGB and VA.

(A) MGB

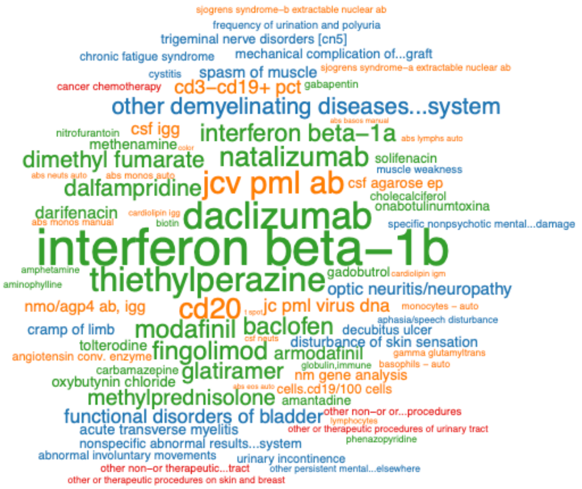

(B) VA

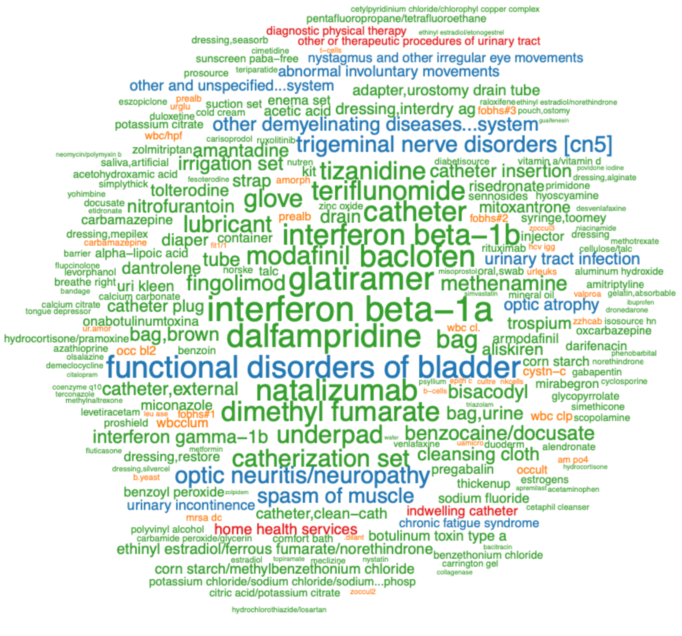

Supplementary Figure 8. KESER selected features for Ulcerative colitis from VA.

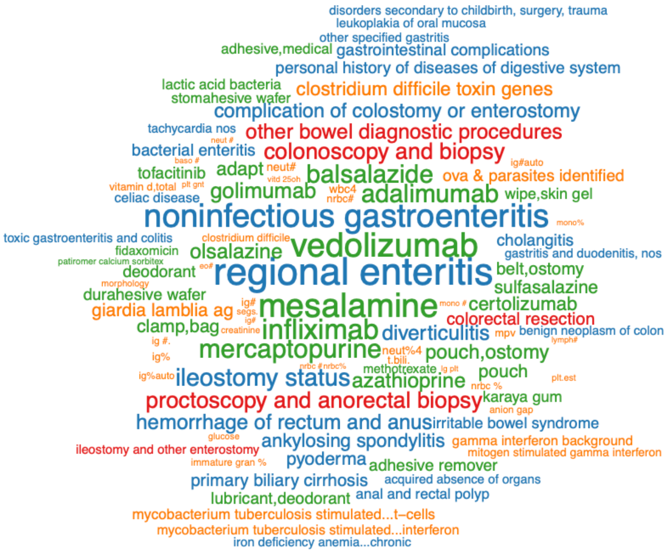

Supplementary Figure 9. KESER selected features for Regional enteritis from MGB and VA.

(A) MGB

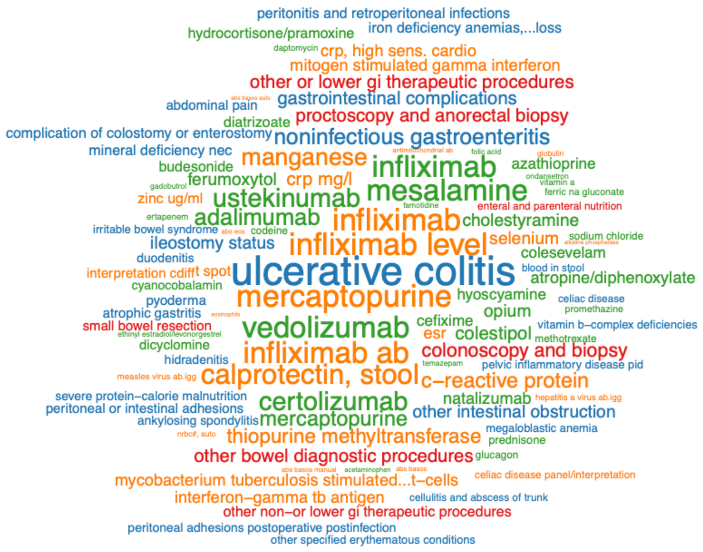

(B) VA

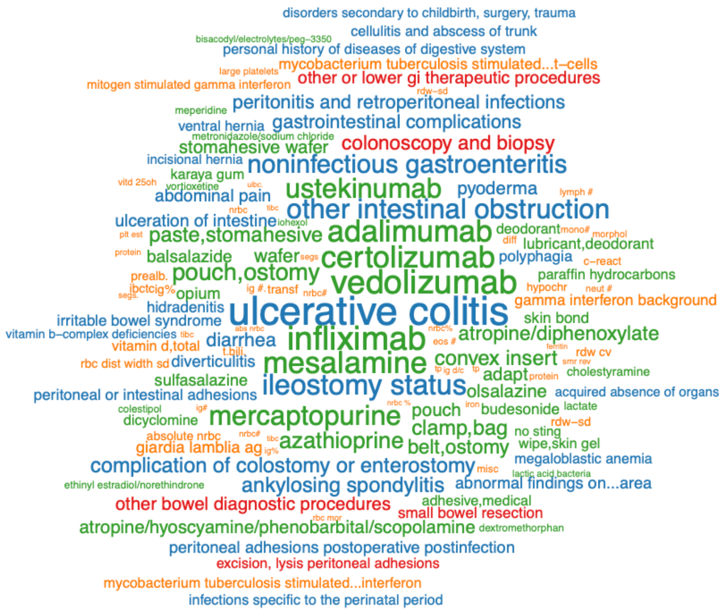

Supplementary Figure 10. Comparison of AUCROCs, AUCPRCs and F-scores with gold standard labels for random forest phenotyping algorithms for 8 diseases using the main PheCode only (PheCode), all features (FULL), SAFE selected features (SAFE), $KESER_{INT}$ selected features using SVD-SPPMI embeddings, $KESER_{MGB}$ selected features using SVD-SPPMI embeddings, as well as $KESER_{INT}$ and $KESER_{MGB}$ selected features based on GloVE embeddings. F-scores are calculated at the cutoff points with the estimated prevalence equal to the population prevalence. The 95% confidence intervals are calculated using bootstrap.

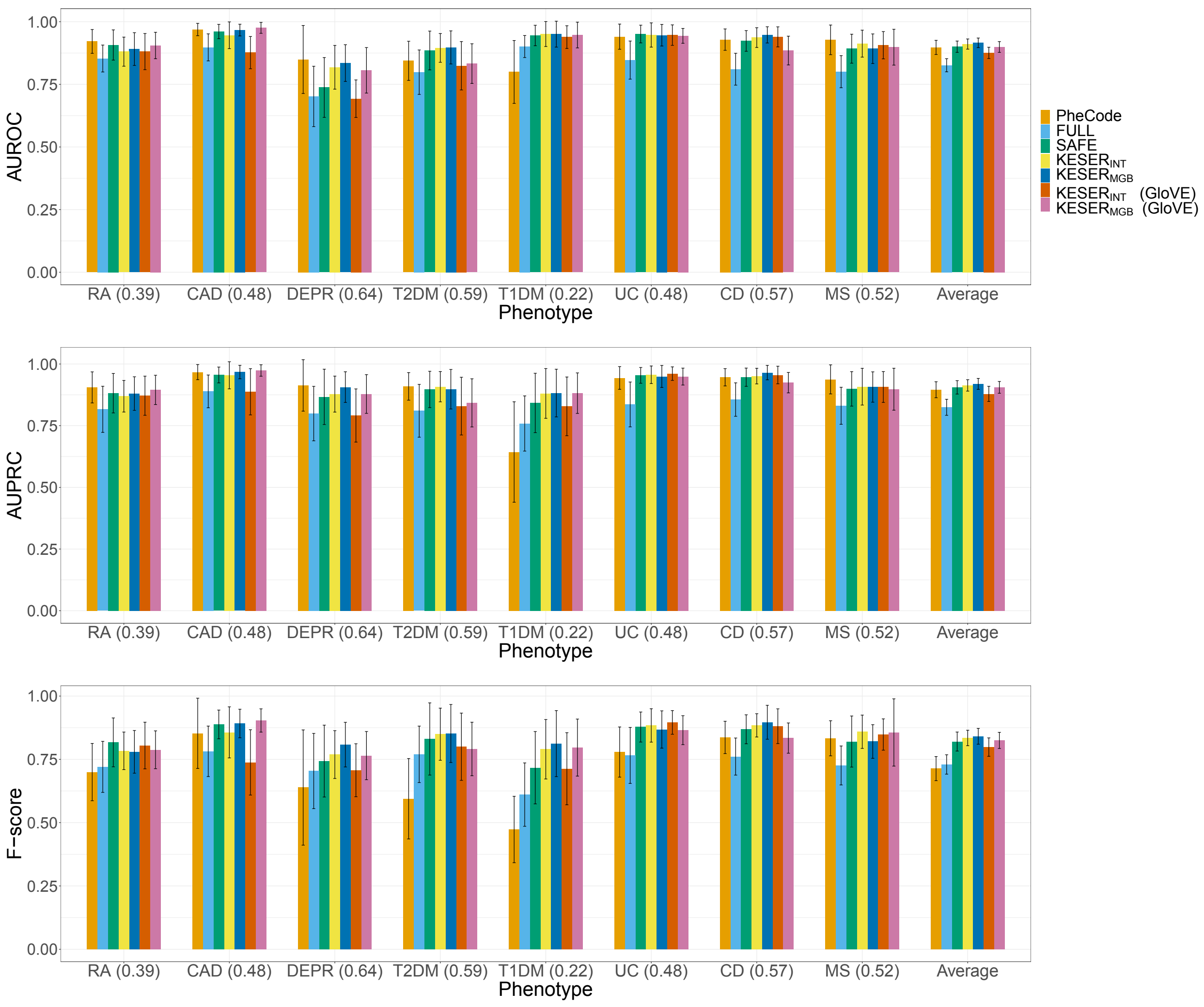

Supplementary Figure 11. Features with high cosine similarity with COVID ICD code.

(a) MGB

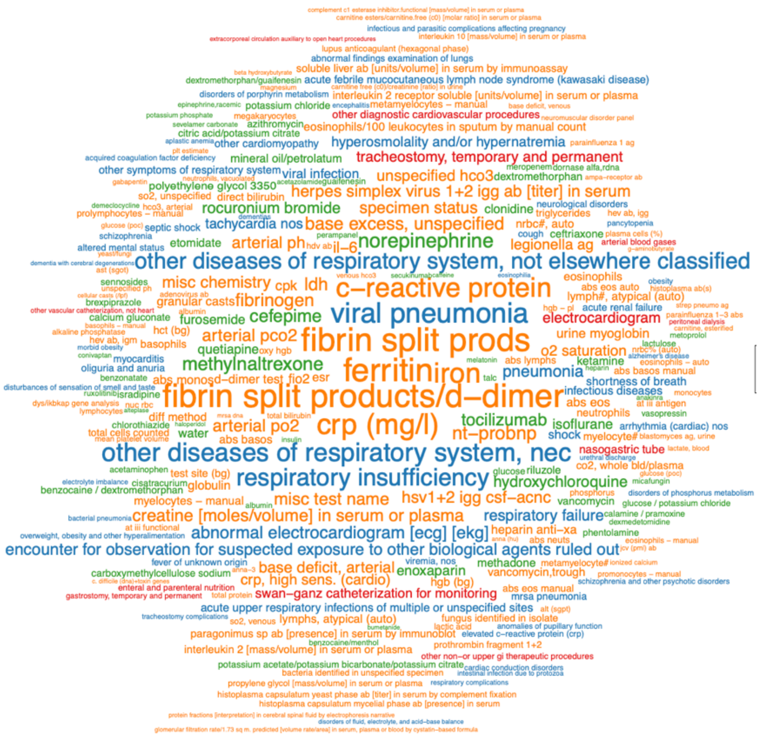

(b) VA.

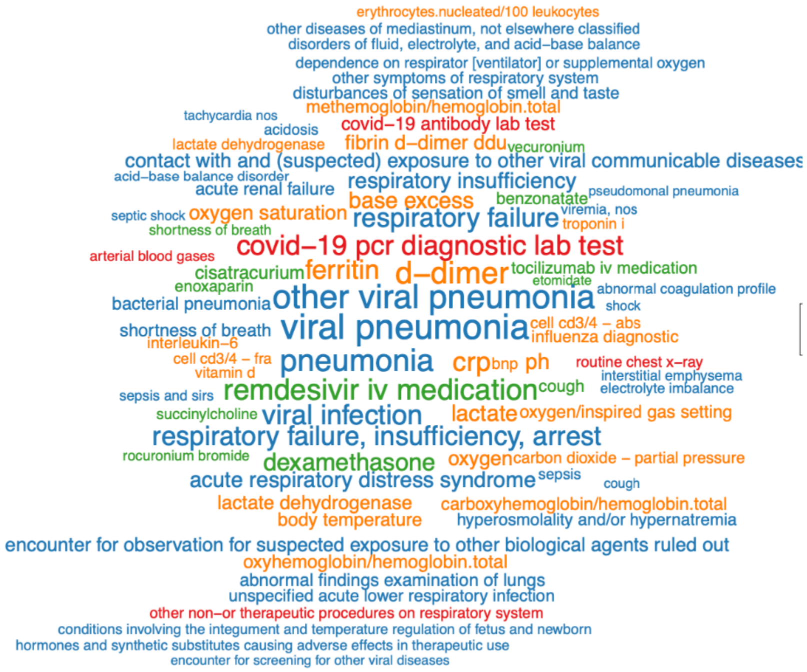

Supplementary Table 1. Known relation pairs from different resources.

Usage	Entity Pairs	Source	Relation Type	# Pairs MGB	# Pairs VA
**Optimize dimension for similarity**	**PheCode-PheCode**	**PheCode hierarchy**	**Similar**	**4220**	**4094**
**Evaluation**	**PheCode-PheCode**	**Wikipedia**	**Related**	**2306**	**2430**
			May Causes	*243*	*258*
			Complications	*435*	*449*
			Symptoms	*549*	*560*
			Risk Factors	*337*	*342*
			Differential Diagnosis	*439*	*499*
			Other	*90*	*99*
	**PheCode-RxNorm**	**MEDRT, SNOMED-CT, Drug.com**	**Related**	**4416**	**4627**
	**RxNorm-RxNorm**	**SNOMED-CT**	**Similar**	**4002**	**3647**
	**Lab-Lab**	**Manual annotated pairs**	**Similar**	**652**	**426**

Supplementary Table 2. Sensitivity analysis for choosing dimension *d*, window size *w* and shift parameter *k*.

Relation Type	*d*	*w*	AUC *k=1*	AUC *k=5*	AUC *k=10*	TPR (FPR=0.01) *k=1*	TPR (FPR=0.01) *k=5*	TPR (FPR=0.01) *k=10*	TPR (FPR=0.05) *k=1*	TPR (FPR=0.05) *k=5*	TPR (FPR=0.05) *k=10*	TPR (FPR=0.1) *k=1*	TPR (FPR=0.1) *k=5*	TPR (FPR=0.1) *k=10*
Similar	100	7	0.849	0.816	0.796	0.351	0.313	0.371	0.567	0.546	0.564	0.681	0.672	0.632
		30	0.857	0.832	0.79	0.385	0.336	0.392	0.573	0.549	0.526	0.683	0.661	0.607
		60	0.861	0.823	0.794	0.37	0.351	0.366	0.583	0.567	0.528	0.694	0.654	0.59
	500	7	0.845	0.817	0.801	0.423	0.452	0.4	0.628	0.651	0.612	0.71	0.705	0.675
		30	0.859	0.821	0.8	0.425	0.418	0.437	0.615	0.637	0.607	0.71	0.699	0.665
		60	0.862	0.825	0.783	0.454	0.465	0.456	0.621	0.638	0.594	0.711	0.704	0.644
	$d_{95\%}$	7	0.828	0.788	0.764	0.394	0.361	0.359	0.586	0.616	0.602	0.683	0.675	0.653
		30	0.832	0.787	0.76	0.396	0.301	0.418	0.577	0.603	0.582	0.673	0.662	0.631
		60	0.841	0.797	0.752	0.442	0.448	0.446	0.594	0.609	0.564	0.677	0.676	0.615
Related	100	7	0.794	0.739	0.7	0.1	0.094	0.131	0.357	0.322	0.294	0.511	0.454	0.399
		30	0.798	0.742	0.707	0.103	0.086	0.089	0.341	0.316	0.261	0.522	0.455	0.365
		60	0.799	0.731	0.696	0.119	0.119	0.133	0.372	0.318	0.26	0.53	0.429	0.362
	500	7	0.802	0.722	0.68	0.165	0.174	0.134	0.439	0.376	0.333	0.556	0.484	0.422
		30	0.804	0.711	0.68	0.14	0.15	0.134	0.408	0.363	0.329	0.557	0.459	0.421
		60	0.805	0.712	0.681	0.16	0.161	0.167	0.421	0.359	0.32	0.563	0.458	0.408
	$d_{95\%}$	7	0.812	0.712	0.673	0.196	0.2	0.181	0.467	0.388	0.342	0.592	0.486	0.432
		30	0.814	0.71	0.678	0.158	0.177	0.148	0.446	0.383	0.322	0.583	0.46	0.418
		60	0.813	0.708	0.678	0.187	0.177	0.175	0.446	0.373	0.326	0.579	0.455	0.411

Supplementary Table 3. Code translation accuracy for VA medication code → RXNORM and PheCode → PheCode using embedding dimensions either optimized for AUC (d_{auc} = 400 at VA, 200 at MGB) or for SNR (d_{snr} = 1800 at VA, 1000 at MGB).

Mapping		Dimensions		Top1	Top5	Top10
VA	MGB	VA	MGB			
VA medication code	RxNorm	400	200	0.382	0.665	0.777
		1800	1000	0.394	0.669	0.793
PheCode	PheCode	400	200	0.421	0.735	0.844
		1800	1000	0.385	0.717	0.806

Supplementary Table 4. AUC and sensitivity at FPR = 0.01, 0.05 and 0.1 of cosine similarity in detecting (a) known similar pairs (RxNorm-RxNorm and Lab-Lab); and (b) related pairs across different types of relationships.

(a) Similar pairs

Entity Pair	Dimension	Method	AUC		Sensitivity					
					FPR = 0.01		FPR = 0.05		FPR = 0.1	
			MGB	VA	MGB	VA	MGB	VA	MGB	VA
RxNorm-RxNorm	50	GloVe	0.790	0.757	0.180	0.146	0.363	0.344	0.470	0.477
		SVD-SPPMI	0.736	0.769	0.107	0.150	0.281	0.291	0.383	0.413
	100	GloVe	0.797	0.763	0.177	0.142	0.350	0.341	0.460	0.460
		SVD-SPPMI	0.749	0.793	0.110	0.191	0.326	0.328	0.439	0.474
	150	GloVe	0.631	0.523	0.045	0.015	0.160	0.059	0.272	0.125
		SVD-SPPMI	0.763	0.804	0.127	0.197	0.359	0.365	0.466	0.516
	500	SVD-SPPMI	0.775	0.841	0.210	0.298	0.430	0.520	0.541	0.638
	d_{snr} (1000,1800)	SVD-SPPMI	0.775	0.830	0.223	0.247	0.430	0.457	0.533	0.602
	d_{auc}(300,500)	SVD-SPPMI	0.773	0.841	0.174	0.298	0.396	0.520	0.533	0.638
	$d_{95\%}$(1800,2900)	SVD-SPPMI	0.772	0.825	0.202	0.242	0.424	0.449	0.541	0.585
Lab-Lab	50	GloVe	0.949	0.962	0.670	0.627	0.844	0.897	0.902	0.932
		SVD-SPPMI	0.913	0.892	0.637	0.270	0.748	0.519	0.819	0.653
	100	GloVe	0.955	0.947	0.689	0.641	0.877	0.836	0.903	0.899
		SVD-SPPMI	0.913	0.922	0.670	0.345	0.791	0.669	0.813	0.817
	150	GloVe	0.903	0.508	0.627	0.007	0.750	0.068	0.801	0.103
		SVD-SPPMI	0.906	0.921	0.686	0.540	0.787	0.775	0.814	0.857
	500	SVD-SPPMI	0.910	0.934	0.724	0.507	0.785	0.838	0.804	0.906
	d_{snr} (1000,1800)	SVD-SPPMI	0.900	0.910	0.724	0.568	0.775	0.805	0.807	0.873
	d_{auc}(300,500)	SVD-SPPMI	0.905	0.934	0.736	0.507	0.790	0.838	0.805	0.906
	$d_{95\%}$(1800,2900)	SVD-SPPMI	0.901	0.910	0.729	0.531	0.778	0.826	0.813	0.883

(b) Related pairs

Entity Pair	Dimension	Method	AUC		Sensitivity					
					FPR = 0.01		FPR = 0.05		FPR = 0.1	
			MGB	VA	MGB	VA	MGB	VA	MGB	VA
**PheCode-PheCode**	50	GloVe	0.947	0.838	0.439	0.290	0.745	0.464	0.867	0.594
		SVD-SPPMI	0.885	0.855	0.379	0.291	0.642	0.504	0.679	0.601
May cause	100	GloVe	0.948	0.870	0.396	0.307	0.773	0.522	0.871	0.621
		SVD-SPPMI	0.901	0.868	0.424	0.326	0.654	0.550	0.737	0.628
	150	GloVe	0.821	0.684	0.227	0.123	0.417	0.157	0.568	0.270
		SVD-SPPMI	0.906	0.879	0.424	0.364	0.646	0.585	0.757	0.674
	500	SVD-SPPMI	0.913	0.913	0.519	0.500	0.658	0.702	0.753	0.771
	d_{snr} (1800,2800)	SVD-SPPMI	0.924	0.925	0.502	0.519	0.774	0.694	0.819	0.810
	d_{auc}(1800,2300)	SVD-SPPMI	0.924	0.925	0.502	0.516	0.774	0.698	0.819	0.810
	$d_{95\%}$(1800,2900)	SVD-SPPMI	0.924	0.925	0.502	0.519	0.774	0.694	0.819	0.810
**PheCode-PheCode**	50	GloVe	0.875	0.799	0.331	0.245	0.595	0.361	0.671	0.470
		SVD-SPPMI	0.856	0.780	0.209	0.234	0.492	0.379	0.605	0.443
complications	100	GloVe	0.881	0.825	0.362	0.263	0.554	0.388	0.709	0.538
		SVD-SPPMI	0.859	0.815	0.241	0.241	0.540	0.419	0.648	0.526
	150	GloVe	0.806	0.659	0.163	0.050	0.337	0.110	0.535	0.201
		SVD-SPPMI	0.877	0.830	0.269	0.314	0.591	0.450	0.690	0.546
	500	SVD-SPPMI	0.880	0.870	0.257	0.303	0.628	0.566	0.726	0.655
	d_{snr} (1800,2800)	SVD-SPPMI	0.884	0.865	0.248	0.285	0.653	0.521	0.743	0.637
	d_{auc}(1800,2300)	SVD-SPPMI	0.884	0.865	0.248	0.285	0.653	0.532	0.743	0.637
	$d_{95\%}$(1800,2900)	SVD-SPPMI	0.884	0.866	0.248	0.287	0.653	0.521	0.743	0.637
**PheCode-PheCode**	50	GloVe	0.894	0.814	0.234	0.217	0.534	0.460	0.723	0.519
		SVD-SPPMI	0.890	0.860	0.339	0.371	0.537	0.505	0.687	0.620
differential diagnosis	100	GloVe	0.889	0.854	0.175	0.278	0.558	0.513	0.727	0.593
		SVD-SPPMI	0.901	0.887	0.324	0.364	0.585	0.538	0.741	0.661
	150	GloVe	0.794	0.672	0.169	0.109	0.311	0.159	0.488	0.281
		SVD-SPPMI	0.910	0.899	0.342	0.366	0.639	0.579	0.754	0.698
	500	SVD-SPPMI	0.896	0.912	0.388	0.461	0.678	0.632	0.754	0.770
	d_{snr} (1800,2800)	SVD-SPPMI	0.898	0.910	0.419	0.482	0.670	0.714	0.750	0.766
	d_{auc}(1800,2300)	SVD-SPPMI	0.898	0.909	0.419	0.471	0.670	0.696	0.750	0.773
	$d_{95\%}$(1800,2900)	SVD-SPPMI	0.898	0.909	0.419	0.482	0.670	0.714	0.750	0.768
**PheCode-PheCode**	50	GloVe	0.834	0.744	0.175	0.147	0.421	0.349	0.598	0.451
		SVD-SPPMI	0.746	0.671	0.098	0.099	0.285	0.211	0.430	0.254

risk factors	100	GloVe	0.836	0.775	0.194	0.144	0.448	0.373	0.607	0.469
		SVD-SPPMI	0.760	0.687	0.092	0.096	0.329	0.211	0.445	0.272
	150	GloVe	0.766	0.623	0.109	0.016	0.279	0.112	0.404	0.197
		SVD-SPPMI	0.772	0.693	0.086	0.114	0.338	0.196	0.439	0.301
	500	SVD-SPPMI	0.806	0.742	0.148	0.137	0.457	0.278	0.531	0.395
	d_{snr} (1800,2800)	SVD-SPPMI	0.816	0.760	0.160	0.184	0.463	0.336	0.611	0.442
	d_{auc}(1800,2300)	SVD-SPPMI	0.816	0.762	0.160	0.181	0.463	0.342	0.611	0.442
	$d_{95\%}$(1800,2900)	SVD-SPPMI	0.816	0.759	0.160	0.184	0.463	0.336	0.611	0.442
**PheCode-PheCode**	50	GloVe	0.870	0.804	0.278	0.210	0.520	0.361	0.701	0.513
		SVD-SPPMI	0.850	0.816	0.121	0.154	0.339	0.361	0.542	0.527
symptoms	100	GloVe	0.869	0.834	0.264	0.203	0.540	0.431	0.703	0.574
		SVD-SPPMI	0.860	0.849	0.134	0.198	0.358	0.421	0.649	0.567
	150	GloVe	0.746	0.602	0.140	0.053	0.313	0.103	0.408	0.167
		SVD-SPPMI	0.870	0.865	0.130	0.196	0.405	0.447	0.638	0.603
	500	SVD-SPPMI	0.874	0.877	0.139	0.204	0.412	0.545	0.706	0.669
	d_{snr} (1800,2800)	SVD-SPPMI	0.881	0.878	0.212	0.234	0.531	0.559	0.745	0.697
	d_{auc}(1800,2300)	SVD-SPPMI	0.881	0.877	0.212	0.228	0.531	0.573	0.745	0.699
	$d_{95\%}$(1800,2900)	SVD-SPPMI	0.881	0.878	0.212	0.234	0.531	0.561	0.745	0.699
**PheCode-RxNorm**	50	GloVe	0.859	0.804	0.240	0.158	0.496	0.374	0.608	0.506
		SVD-SPPMI	0.827	0.773	0.167	0.148	0.442	0.316	0.585	0.434
	100	GloVe	0.863	0.817	0.254	0.217	0.493	0.421	0.625	0.533
		SVD-SPPMI	0.840	0.802	0.179	0.198	0.499	0.398	0.622	0.528
	150	GloVe	0.717	0.590	0.058	0.019	0.221	0.102	0.352	0.196
		SVD-SPPMI	0.846	0.822	0.250	0.219	0.536	0.440	0.655	0.574
	500	SVD-SPPMI	0.848	0.839	0.352	0.330	0.584	0.557	0.678	0.652
	d_{snr} (1800,2800)	SVD-SPPMI	0.850	0.853	0.435	0.379	0.608	0.600	0.685	0.697
	d_{auc}(1800,2300)	SVD-SPPMI	0.850	0.853	0.435	0.378	0.608	0.600	0.685	0.696
	$d_{95\%}$(1800,2900)	SVD-SPPMI	0.850	0.853	0.435	0.378	0.608	0.601	0.685	0.696

Supplementary Table 5. AUCs and sensitivity at FPR = 0.01, 0.05 and 0.10 of between-vector cosine similarity in detecting known similar pairs (RxNorm-RxNorm and Lab-Lab) and related pairs (PheCode-PheCode; PheCode-RxNorm) with embeddings trained via SVD-SPPMI at different choices of dimensions d.

Relation Type	Embedding		AUC		Sensitivity					
					FPR=0.01		FPR=0.05		FPR=0.1	
	d	Method	MGB	VA	MGB	VA	MGB	VA	MGB	VA
Similar	50	GloVe	0.869	0.86	0.425	0.386	0.603	0.62	0.686	0.704
		SVD-SPPMI	0.825	0.831	0.372	0.21	0.515	0.405	0.601	0.533
	100	GloVe	0.876	0.855	0.433	0.391	0.614	0.588	0.681	0.68
		SVD-SPPMI	0.831	0.857	0.39	0.268	0.559	0.499	0.626	0.646
	150	GloVe	0.767	0.515	0.336	0.011	0.455	0.064	0.536	0.114
		SVD-SPPMI	0.835	0.862	0.406	0.368	0.573	0.570	0.64	0.686
	500	GloVe	0.503	0.514	0.04	0.006	0.108	0.040	0.183	0.102
		SVD-SPPMI	0.842	0.888	0.467	0.403	0.607	0.679	0.673	0.772
	1000	GloVe	0.526	0.507	0.051	0.013	0.115	0.047	0.225	0.093
		SVD-SPPMI	0.837	0.882	0.473	0.418	0.602	0.648	0.67	0.764
Related	50	GloVe	0.873	0.805	0.275	0.198	0.538	0.384	0.659	0.505
		SVD-SPPMI	0.844	0.789	0.189	0.164	0.456	0.351	0.604	0.463
	100	GloVe	0.876	0.828	0.286	0.247	0.542	0.435	0.672	0.558
		SVD-SPPMI	0.854	0.817	0.205	0.197	0.498	0.41	0.647	0.538
	150	GloVe	0.758	0.623	0.126	0.053	0.302	0.128	0.438	0.217
		SVD-SPPMI	0.862	0.833	0.236	0.202	0.54	0.442	0.671	0.57
	500	GloVe	0.630	0.625	0.065	0.047	0.155	0.112	0.24	0.199
		SVD-SPPMI	0.864	0.854	0.304	0.291	0.589	0.543	0.705	0.66
	1000	GloVe	0.639	0.622	0.055	0.054	0.147	0.128	0.244	0.207
		SVD-SPPMI	0.866	0.859	0.332	0.311	0.606	0.573	0.710	0.675

Supplementary Consortium Member list:

VA Million Veteran Program: Core Acknowledgement for Publications

Samuel Aguayo[10], Sunil Ahuja[11], Dean P. Argyres[12], Mihaela Aslan[8], Zuhair Ballas[13], Jean Beckham[14,] Sujata Bhushan[15], Edward Boyko[16], James Breeling[17], Jessica V. Brewer[2], Mary T. Brophy[2], Juan P. Casas[2], Kyong-Mi Chang[18], Lori Churby[19], David Cohen[20], Todd Connor[12], Joseph Constans[21], Louis Dellitalia[22], Nhan Do[2], Scott DuVall[23], Joseph Fayad[24], Hermes Florez[25], Melinda Gaddy[26], Saib Gappy[27], J. Michael Gaziano[2], Gretchen Gibson[28], Michael Godschalk[29], Jennifer Greco[30], Samir Gupta[31], Salvador Gutierrez[32], Kimberly Hammer[33], Mark Hamner[34], John Harley[35], Elizabeth Hauser[14], Grant Huang[17], Donald E. Humphries[2], Adriana Hung[36], Robin Hurley[37], Mostaqul Huq[38], Pran Iruvanti[39], Douglas Ivins[40], Frank Jacono[41], Darshana Jhala[18], Laurence Kaminsky[42], Scott Kinlay[2], Jon Klein[43], Suthat Liangpunsakul[44], Jack Lichy[45], Stephen Mastorides[46], Roy Mathew[47], Kristin Mattocks[48], Rachel McArdle[49], Laurence Meyer[24], Paul Meyer[50], Jonathan Moorman[51], Timothy Morgan[52], Jennifer Moser[17], Sumitra Muralidhar[17], Maureen Murdoch[53], Christopher J. O'Donnell[2], Olaoluwa Okusaga[54], Kris Ann Oursler[55], Saiju Pyarajan[2], Rachel Ramoni[17], Nora Ratcliffe[56], Michael Rauchman[57], Brooks Robey[58], George Ross[59], Luis E. Selva[2], Richard Servatius[60], Satish Sharma[61], Shahpoor Shayan[2], Scott Sherman[62], Peruvemba Sriram[63], Todd Stapley[64], Brady Stephens[65], Robert Striker[66], Patrick Strollo[67], Yan Sun[68], Neeraj Tandon[69], Philip S. Tsao[19], Gerardo Villareal[12], Agnes Wallbom[70], John Wells[71], Stacey B. Whitbourne[2], Jeffrey Whittle[72], Mary Whooley[73], Peter Wilson[68], Junzhe Xu[74], Shing Shing Yeh[75], Hongyu Zhao[8]

Affiliations

[10]*Phoenix VA Health Care System; Phoenix, AZ, USA*

[11]*South Texas Veterans Health Care System; San Antonio, TX, USA*

[12]*New Mexico VA Health Care System; Albuquerque, NM, USA*

[13]*Iowa City VA Health Care System; Iowa City, IA, USA*

[14]*Durham VA Medical Center, Durham, NC, USA*

[15]*VA North Texas Health Care System; Dallas, TX, USA*

[16]*VA Puget Sound Health Care System; Seattle, WA, USA*

[17]*US Department of Veterans Affairs; Washington, DC, USA*

[18]*Philadelphia VA Medical Center; Philadelphia, PA, USA*

[19]*VA Palo Alto Health Care System; Palo Alto, CA, USA*

[20]*Portland VA Medical Center; Portland, OR, USA*

[21]*Southeast Louisiana Veterans Health Care System; New Orleans, LA, USA*

[22]*Birmingham VA Medical Center; Birmingham, AL, USA*

[23]*VA Salt Lake City Health Care System; Salt Lake City, UT, USA*

[24]*VA Southern Nevada Healthcare System; North Las Vegas, NV, USA*

[25]*Miami VA Health Care System; Miami, FL, USA*

[26]*VA Eastern Kansas Health Care System; Leavenworth, KS, USA*

[27]*John D. Dingell VA Medical Center; Detroit, MI, USA*

[28]*Veterans Health Care System of the Ozarks; Fayetteville, AR, USA*
[29]*Richmond VA Medical Center; Richmond, VA, USA*
[30]*Sioux Falls VA Health Care System; Sioux Falls, SD, USA*
[31]*VA San Diego Healthcare System; San Diego, CA, USA*
[32]*Edward Hines, Jr. VA Medical Center; Hines, IL, USA*
[33]*Fargo VA Health Care System; Fargo, ND, USA*
[34]*Ralph H. Johnson VA Medical Center; Charleston, SC, USA*
[35]*Cincinnati VA Medical Center; Cincinnati, OH, USA*
[36]*VA Tennessee Valley Healthcare Slystem; South Nashville, TN, USA*
[37]*W.G. (Bill) Hefner VA Medical Center; Salisbury, NC, USA*
[38]*VA Sierra Nevada Health Care System; Reno, NV, USA*
[39]*Hampton VA Medical Center; Hampton, VA, USA*
[40]*Eastern Oklahoma VA Health Care System; Muskogee, OK, USA*
[41]*VA Northeast Ohio Healthcare System; Cleveland, OH, USA*
[42]*VA Health Care Upstate New York; Albany, NY, USA*
[43]*Louisville VA Medical Center; Louisville, KY, USA*
[44]*Richard Roudebush VA Medical Center; Indianapolis, IN, USA*
[45]*Washington DC VA Medical Center; Washington, D. C., USA*
[46]*James A. Haley Veterans' Hospital; Tampa, FL, USA*
[47]*Columbia VA Health Care System; Columbia, SC, USA*
[48]*Central Western Massachusetts Healthcare System; Leeds, MA, USA*
[49]*Bay Pines VA Healthcare System; Bay Pines, FL, USA*
[50]*Southern Arizona VA Health Care System; Tucson, AZ, USA*
[51]*James H. Quillen VA Medical Center; Mountain Home, TN, USA*
[52]*VA Long Beach Healthcare System; Long Beach, CA, USA*
[53]*Minneapolis VA Health Care System; Minneapolis, MN, USA*
[54]*Michael E. DeBakey VA Medical Center; Houston, TX, USA*
[55]*Salem VA Medical Center; Salem, VA, USA*
[56]*Manchester VA Medical Center; Manchester, NH, USA*
[57]*St. Louis VA Health Care System; St. Louis, MO, USA*
[58]*White River Junction VA Medical Center; White River Junction, VT, USA*
[59]*VA Pacific Islands Health Care System; Honolulu, HI, USA*
[60]*Syracuse VA Medical Center; Syracuse, NY, USA*
[61]*Providence VA Medical Center; Providence, RI, USA*
[62]*VA New York Harbor Healthcare System; New York, NY, USA*

[63] *N. FL/S. GA Veterans Health System; Gainesville, FL, USA*
[64] *VA Maine Healthcare System; Augusta, ME, USA*
[65] *Canandaigua VA Medical Center; Canandaigua, NY, USA*
[66] *William S. Middleton Memorial Veterans Hospital; Madison, WI, USA*
[67] *VA Pittsburgh Health Care System; Pittsburgh, PA, USA*
[68] *Atlanta VA Medical Center; Atlanta, GA, USA*
[69] *Overton Brooks VA Medical Center; Shreveport, LA, USA*
[70] *VA Greater Los Angeles Health Care System; Los Angeles, CA, USA*
[71] *Edith Nourse Rogers Memorial Veterans Hospital; Bedford, MA, USA*
[72] *Clement J. Zablocki VA Medical Center; Milwaukee, WI, USA*
[73] *San Francisco VA Health Care System; San Francisco, CA, USA*
[74] *VA Western New York Healthcare System; Buffalo, NY, USA*
[75] *Northport VA Medical Center; Northport, NY, USA*
